# Hunting indicators for community-led wildlife management in tropical Africa

**DOI:** 10.1038/s44185-024-00048-4

**Published:** 2024-07-18

**Authors:** Davy Fonteyn, Adeline Fayolle, Julia E. Fa, Hadrien Vanthomme, Philippe Vigneron, Cédric Vermeulen, Rémi Malignat, Benoît Konradowski, Mexan Noel Yia Okanabene, Stéphane Axel Dibotty-di Moutsing, Samuel Pereira Dias, Christophe Deniau, Guillaume Cornu, Marion Groschêne, Daniel Cornélis

**Affiliations:** 1 CIRAD, UPR Forêts et Sociétés, Campus International de Baillarguet, Montpellier, France; 2 Forêts et Sociétés, Univ Montpellier, CIRAD, Montpellier, France; 3 Université de Liège – Gembloux Agro-Bio Tech, FORIL, Unité Gestion des Ressources Forestières, Gembloux, Belgium; 4 Department of Natural Sciences, Faculty of Science and Engineering, Manchester Metropolitan University, Manchester, UK; 5 Center for International Forestry Research (CIFOR), Kota Bogor, Jawa Barat Indonesia

**Keywords:** Conservation biology, Sustainability, Conservation biology

## Abstract

Engaging local communities is pivotal for wildlife conservation beyond protected areas, aligning with the 30 × 30 target of the Kunming-Montreal Global Biodiversity Framework. We assessed the effectiveness of 33 offtake indicators, derived from hunter declarations, in monitoring the status and extent of degradation of hunted wildlife sourced from camera trap surveys and faunal composition analysis. The rodents:ungulates ratio in offtake and the mean body mass of total offtake emerged as practical and robust indicators of faunal degradation within hunting systems, with significant potential for broader application in similar tropical forest environments. Our findings provide a blueprint for managing and conserving natural resources in tropical regions through community-based initiatives. Involving local stakeholders ensures sustainable wildlife use and fosters ownership and responsibility. This study advances conservation efforts, bridging scientific rigor with community engagement for effective biodiversity preservation.

## Introduction

In tropical ecosystems, medium- to large-bodied mammals ( > 5 kg) play significant roles as herbivores, seed dispersers, and predators^1^. They impact prey populations and the composition and distribution of the surrounding vegetation^2^. Nonetheless, these mammals are also the primary targets of human hunters^3^.

Research across all tropical regions has consistently demonstrated that overhunting leads to predictable shifts in the composition of forest wildlife communities: larger species tend to disappear first, while their smaller counterparts endure^4^. This is because larger species are slower breeders, are found at lower densities, and are actively targeted by hunters due to their higher meat yields^5^. Their sensitivity is further exacerbated by the increase in illegal wildlife trade to cater to urban centers, as well as habitat loss and fragmentation. Responses such as density compensation, facilitated by the release of competitors and predators^6^, may occur in taxa that are not specifically targeted by hunters or that exhibit some resistance to hunting pressure, making degradation pathways even more complex to grasp.

Assessing the status of wildlife in tropical forests is critical, not only because it disrupts ecosystem functioning^7^ but also because the meat of many animals, particularly medium-sized and large mammals, is vital for the food security and livelihoods of many human communities residing in these forests^8^. This is the case in West and Central African forests, where the exploitation of medium and large mammals is particularly high^3^. Assessing hunting-induced degradation fundamentally requires monitoring of hunting sites to compare with reference states where faunas are better conserved^9,10^. Reference sites should ideally be pristine spaces, such as protected areas; however, even those sites often experience some level of hunting, although this level is arguably lower than that of nonprotected sites.

Indigenous and local communities, especially hunters, are the most knowledgeable users of tropical forest areas and can actively contribute to monitoring the state of the animal populations they target using simple indicators based on their offtake records^11–13^. Published indicators, such as the mean body mass (MBM) index, have been used to determine trends in the exploitation of mammals and birds in West and Central Africa^14^, where a decrease in MBM indicates a reduction in the populations of larger (*i.e*., less resilient) species. Additionally, the correlation between the ratio of hunted rodents to hunted ungulates and human density at source locations, as a proxy for hunter numbers^15^, suggests that rodents (*i.e*., smaller and therefore potentially more resilient species) increase in more hunted areas. Other research has compared the MBM (which correlates with changes in rodents:ungulates ratio) or other indices (*e.g*., population growth rate) with human population density^16,17^. However, these indicators have not been interrelated or validated with independent assessments of the species presence, abundance, and biomass of faunas in more intact forests.

**Fig. 1:**
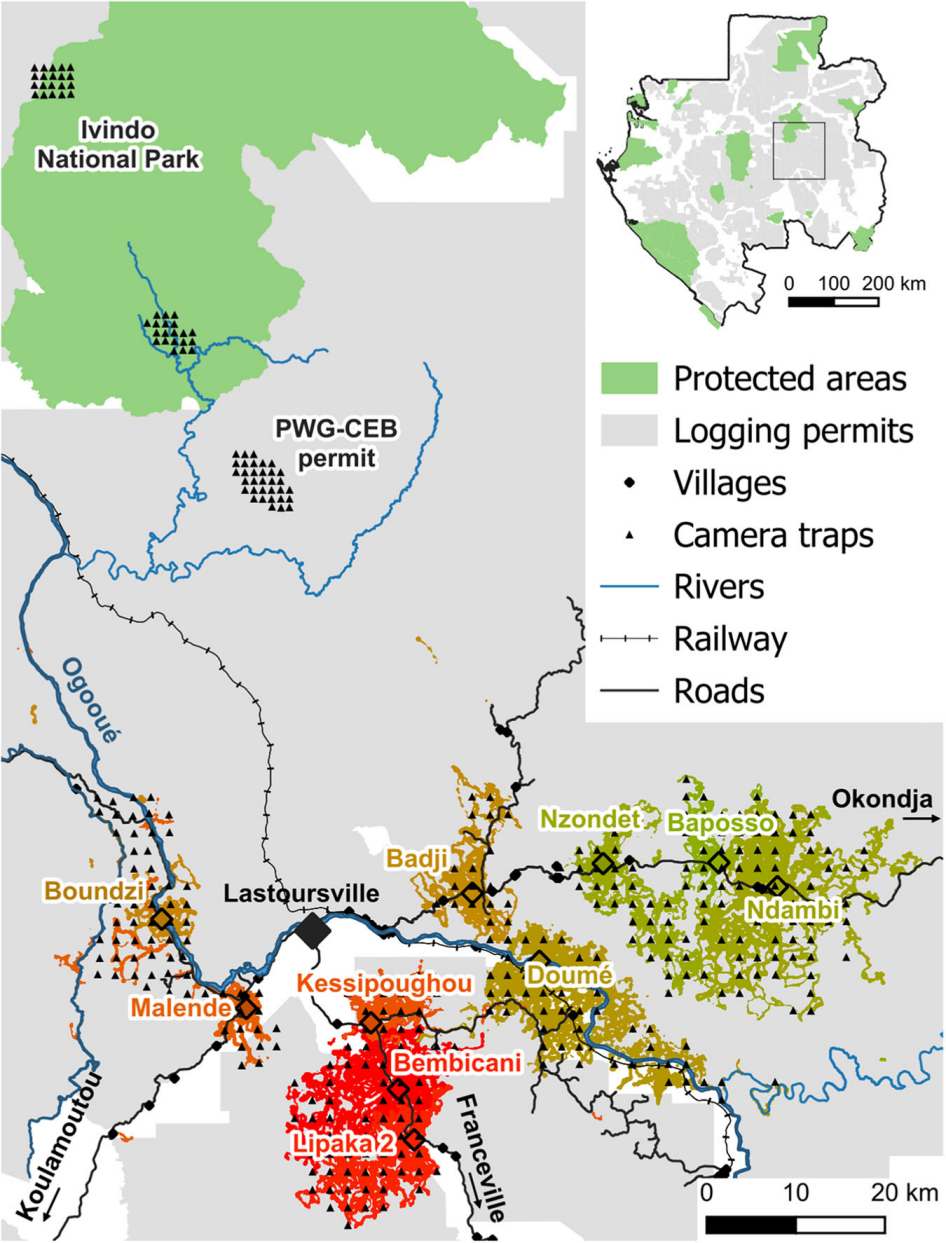
Study sites and partner hunter communities. A total of 314 hunters self-reported their hunts and the animals they killed over a period of 10 to 42 months, depending on the studied hunter communities (empty diamond). Additionally, 75% of these hunters agreed to carry GPS to record hunting trips. Hunting tracks were used to map the territories shared by all hunters from the same hunter community using movement-based kernel analysis (see Methods). The colors of each hunting territory denote the degree of faunal degradation compared to the baseline faunal assemblage of the Ivindo National Park (see Fig. 2 and Supplementary Table 1). Camera trap arrays (black triangles), roads (black lines) and railways (dashed), and the Ogooué River and its affluents (blue lines) are also indicated across the studied area in Gabon.

Vast tropical moist forests cover 85% of Gabon, most of which are relatively undisturbed wildernesses^18^. This intactness owes much to the country’s relatively sparse human population. Nevertheless, even though deforestation is not a substantial threat^19^, hunting for wild meat consumption remains a widespread practice. Here, we drew upon field data collected by 314 hunters from 10 hunter communities near Lastoursville in eastern Gabon (Fig. 1) for 42 months; these hunters represented a total of 12,970 hunting trips (Supplementary Table 2). Using systematic camera trapping (CT) surveys totaling 374 camera traps and 17,500 camera days, we assessed the composition and relative abundance of terrestrial and semiterrestrial forest species. This ranged from small murids and land birds to forest elephants. CT surveys were used to sample all 10 community hunting territories, as well as the main reference site, the adjacent Ivindo National Park (NP) and a nearby logging concession granted to the *Precious Woods Gabon*-*Compagnie Equatoriale des Bois* (PWG-CEB), in a sector that has been inaccessible and unlogged since 2008. Using the Bray‒Curtis (BC) dissimilarity index and CT survey results, we quantified the faunal degradation of terrestrial species assemblages in all hunting territories by comparing their species composition with that of the reference species assemblage in the Ivindo NP. Additionally, we calculated 33 hunting offtake indicators using participatory hunting records, which included tallying and weighing hunter harvests and tracking hunting trips via GPS technology. By correlating each offtake indicator with the BC index, we identified those indicators that are strongly linked to the status of hunted wildlife populations. Our results enhance our understanding of hunting’s impact on local wildlife and identify indicators for future research and for conservation and restoration efforts, applicable not only in tropical Africa but also in similar tropical forest environments.

## Results

### Assessing faunal degradation

**Fig. 2:**
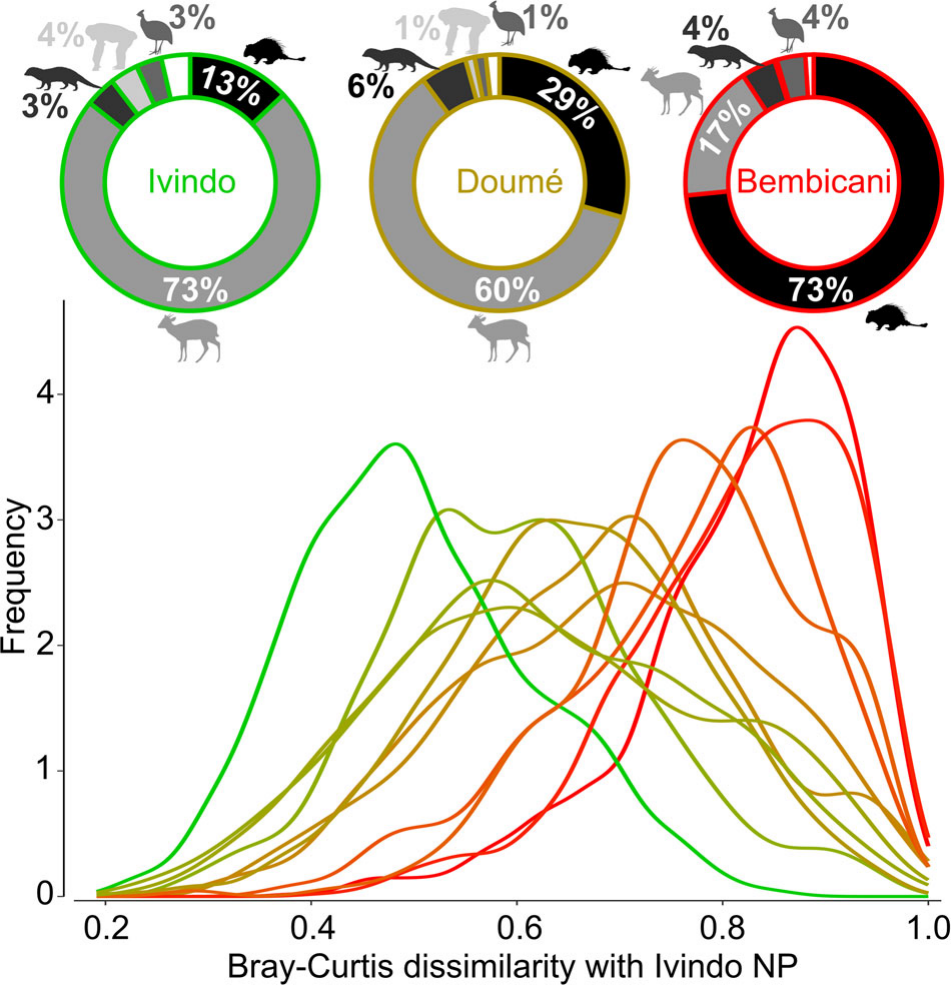
Quantitative assessment of the degree of faunal degradation using the Bray‒Curtis dissimilarity index. The faunal composition of each camera trap (CT) within a hunting territory (*N* = 301 CTs) was individually compared with that of each camera trap (*N* = 38 CTs) at the reference site, Ivindo National Park (NP), using the Bray‒Curtis (BC) dissimilarity index. The distribution of the 11,438 dissimilarity values (301 × 38) is presented with a smoothed histogram per hunter community. A donut chart showing the proportions of CT detections of each major species group (rodents, ungulates, carnivores, apes, terrestrial birds and other species) is displayed for the Ivindo NP, Doumé (BC dissimilarity median = 0.66) and Bembicani (BC dissimilarity median = 0.85) study sites.

Camera trap (CT) data were used to examine the faunal composition of all study sites (see Methods, Supplementary Table 1). We conducted a quantitative comparison, using the Bray-Curtis (BC) dissimilarity index (see Methods), to assess changes in faunal composition within hunted sites compared to the baseline faunal assemblage of the Ivindo National Park (NP), enabling us to quantify the degree of faunal degradation within the hunting territory of each hunter community^9^. Higher values (close to 1) indicate greater dissimilarity with the Ivindo NP and, by extension, more severe faunal degradation. All the sites were then ranked and colored according to the median BC index (Fig. 2). Species richness did not significantly vary along the faunal degradation gradient (Supplementary Fig. 1a); however, there was a meaningful difference in the contributions of various animal taxa to the total number of detection events (Supplementary Fig. 1b). At more degraded sites (high BC index), rodents dominated CT detection—up to 76%—while at Ivindo NP, their contribution decreased to less than 13% (Supplementary Table 1). Additionally, there was an inversely proportional decline in ungulates, particularly for blue duikers (*Philantomba monticola*) and medium-sized ungulates, which included *Cephalophus* duikers and the water chevrotain (*Hyemoschus aquaticus*). Larger ungulates, particularly the red river hog (*Potamochoerus porcus*), which is an important source of meat, were less frequently detected in general but also experienced a significant decline in more degraded sites (Supplementary Fig. 2).

### Hunter offtake and indicators of faunal degradation

Offtake surveys revealed different hunting strategies used by the participating hunter communities (Supplementary Table 2) as well as complex interactions between hunter practices and returns (Supplementary Fig. 5). Although there was variability among hunters in terms of hunting methods, 12-gauge shotguns accounted for approximately 78% of all animals harvested. Trapping was responsible for approximately 19% of the hunter kills, while a smaller fraction of the animals were caught by hand. Hunting with firearms took place at night and during the day, with an equal distribution of hunting hours between the two. However, 71% of the animals killed with guns were shot at night. Gun hunts typically lasted for an average of 14 hours, with only some overnight camps. Consequently, hunting activities were concentrated close to the hunters’ settlements. Approximately 95% and 50% of the hunting activities occurred below average distances of 10.1 and 2.9 km from an access road or river, respectively. Overall, the most hunted animals were ungulates, with the blue duiker (25% of the harvest), Peters’ duiker (*Cephalophus callipygus*; 13%), bay duiker (*C. dorsalis*; 9%), and red river hog (4%), together comprising up to 65% of the total harvested biomass (Supplementary Fig. 4). Among the rodents, brush-tailed porcupine represented 20% of all kills and 7% of the total biomass. Cash transactions involved approximately 70% of all hunted animals.

**Fig. 3:**
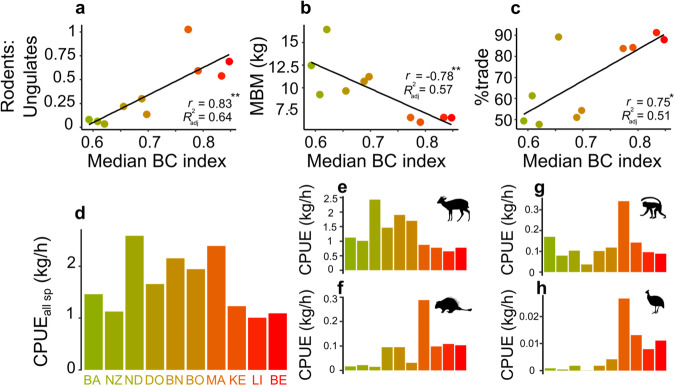
Hunting offtake indicators in relation to the degree of faunal degradation. Village faunal degradation is represented by the median value for each hunter community of the Bray-Curtis (BC) dissimilarities with the reference site. Trends of offtake indicators related to **a** the ratio of rodent kills to ungulate kills, **b** the mean body mass (MBM) of the total offtake and **c** the percentage of pieces traded are represented along the degradation gradient. The Pearson correlation coefficient (*r*) and the coefficient of determination (R^2^) of linear regressions of each combination are displayed. The mean biomass harvested in kg per hunting hour (catch-per-unit-effort metric) is also provided for all species **d** and for each species group: ungulates **e**, rodents **f**, primates **g**, and birds **h**. The villages are organized in the bar charts by increasing faunal degradation (Supplementary Table 1), BA stands for Baposso, NZ for Nzondet, ND for Ndambi, DO for Doumé, BN for Bakoussou-Ndekabalandji, BO for Boundzi, MA for Malende, KE for Kessipoughou, LI for Lipaka2 and BE for Bembicani.

We computed a suite of 33 different indicators from the hunting offtake data and hunter GPS self-follows: seven related to the hunting method, six related to the catch species composition, 19 related to hunter returns and one related to the trade of wild meat (Supplementary Table 3). Seven of these offtake indicators were significantly related to faunal degradation (Fig. 3, Supplementary Table 4), namely: (1) percent of rodents in offtake, *r* = 0.88, *p* = 0.001; (2) percent ungulates in offtake, *r* = -0.886; *p* = 0.001; (3) mean body mass (kg) of the total offtake, *r* = -0.784, *p* = 0.007; (4) percent of pieces traded, *r* = 0.753, *p* = 0.012; (5) rodent biomass per hunting kilometer (kg/km), *r* = 0.751, *p* = 0.012; (6) bird biomass per hunting trip (kg/hunt), *r* = 0.745, *p* = 0.013; (7) rodent biomass per hunting trip (kg/hunt), *r* = 0.698, *p* = 0.025. Of these, only the first two were significant after Bonferroni adjustment due to the small statistical power associated with our limited number of hunter communities (n = 10). These two indicators can be used independently as a measure of faunal degradation or combined into a single rodents:ungulates ratio, as used in other studies in West and Central Africa^15,20,21^. This ratio increases significantly along the faunal degradation gradient, from less than 0.1 to more than 1, showing that rodents are proportionally hunted more in areas that are strongly impacted by hunting activities (Fig. 3a). Other important indicators are the mean body mass (MBM) of the total offtake, which decreases as the degree of faunal degradation increases (Fig. 3b), and the percentage of traded animals, which increases significantly with faunal degradation, from 48% to more than 90% in the most degraded hunter communities (Fig. 3c).

Other reported hunting indices^20,22^ were not significantly correlated with faunal degradation (see Supplementary Table 4). Specifically, we did not observe a significant relationship between the percentage of blue duiker in total duiker catches or the annual extraction rate per square kilometer. Catch-per-unit-effort (CPUE) metrics are typically expected to positively correlate with the absolute abundance of a species or a group of species, if effort is appropriately considered^23^. However, in our study, most of the CPUE metrics, except for the ones mentioned earlier, were not correlated with the faunal degradation gradient (see Fig. 3d).

Our examination of the performance of indicators shows that the three indicators shown in Fig. 3a–c are related to the status of hunted wildlife populations. All three, but especially the rodents:ungulates ratio and the mean body mass are simple to calculate and easy to implement through hunter self-monitoring schemes. However, the percentage of traded animals, which is also one of the indicators highly correlated with faunal degradation, could be more complicated to assess under field conditions and is also influenced by additional variables such as shifts in socioeconomic factors and market accessibility^15^.

## Discussion

Hunting-induced shifts in wildlife communities are complex and encompass more than just the mere presence or absence of sensitive species. This study, as well as prior research in the region^24,25^, underscores the contributions of various mechanisms, such as density compensation processes, for which detailed species composition analyses are needed for proper evaluation. Despite presenting a formidable challenge, the routine monitoring of wildlife populations in vast tropical forests such as the Amazon and Congo Basins^26^, where wildlife hunting is common, has become possible thanks to the widespread adoption of innovative wildlife monitoring techniques such as camera traps. Conducting camera trap surveys to document terrestrial animal communities in tropical forests however demands a high level of technical proficiency in configuring equipment and interpreting data. Additionally, this approach necessitates significant logistical support and human resources^27–29^. Because of such demanding requirements, these surveys are not practical for community-led wildlife management. This underscores the necessity of identifying simpler wildlife monitoring methods that local communities can more readily implement. If validated, local forest-dwelling communities could become key players in gathering reliable wildlife data for management purposes, supported by their long-established traditional ecological knowledge. This resonates with the concept of ‘paraecologists’, recognized for decades, where local individuals who have extensive knowledge of their environments can gather research data useful for natural resource management purposes^30^. Although successful at a number of Amazonian research sites^31,32^, such an approach has not yet been widely implemented in sub-Saharan Africa.

Our broad-scale analysis of wildlife exploitation across diverse hunter communities allowed us to represent faunal degradation patterns within hunting territories. Regardless of variations in the selectivity of hunted species, consistent trends emerge from both camera trap and offtake data. Animal communities experiencing lower hunting pressure consist of more ungulates and fewer rodents, as reflected by the rodents:ungulates ratio in offtake and the hunter offtake mean body mass. Both metrics act as proxies of species composition, demonstrating hunters’ tendency to exploit readily available species. The proportion of carcasses traded is also related to faunal degradation, although the market accessibility and socioeconomic conditions of hunter communities could be additional significant determinants^17^. In contrast, indicators such as the catch-per-unit-effort (CPUE) indices are poorly correlated with faunal degradation, limiting their ability to accurately predict the overall status of the hunted species assemblage.

By comparing extensive hunting and camera trap datasets with unmatched spatial and temporal coverage, we have developed two simple yet highly effective indicators for assessing faunal degradation in hunting systems: the rodents-to-ungulates ratio in offtake and the mean body mass of total offtake. Scaling up this approach can offer invaluable insights into regional defaunation patterns, complementing prior large-scale assessments^33,34^. Moreover, these indicators can aid in identifying areas for restoration and delineating relatively pristine sites warranting conservation beyond designated protected areas, in alignment with initiatives like the Global Biodiversity Framework’s “30×30” Target and other effective area-based conservation measures (OECMs), where conservation is predominantly achieved as a by-product of other management^35^.

Proposing two practical and consistent indicators—the ratio of rodents to ungulates and the mean body mass of hunter offtake—our study advocates for their adoption in community-led initiatives within any moist forest in tropical Africa. These indicators, proven effective and simple to calculate, can be easily embraced by hunters and governmental agencies and integrated into hunter self-monitoring programs. While the applicability of these indicators in other African moist forests still needs to be tested further, there is sufficient evidence from other studies^14^ to suggest that our two indicators are likely to be universal.

The two indicators are interconnected and align with the empirical rule linking body mass to breeding potential and density^36–38^. These relationships explain how large-bodied mammals, being slow breeders and naturally occurring in lower numbers, are typically the first to disappear in most tropical hunting systems, ultimately replaced by smaller, faster-breeding, and more abundant species.

While our selected indicators show promise for application in tropical forests around the world, it is important to consider the variations in species assemblages in each region. For example, in the Amazon, non-volant mammals (including species like the lowland tapir, the largest mammal in the Neotropics) are less diverse and consist of species with smaller body size and biomass compared to their counterparts in the Paleotropics^39^. Additionally, the distribution of species between terrestrial and arboreal habitats contrasts dramatically between the Amazon and Congo Basins^39^, a feature which impacts hunting practices e.g., hunters tend to target more arboreal prey (such as atelid and cebid monkeys) in the Amazon than in the Congo Basin^40^. These observations highlight the necessity of integrating arboreal species more thoroughly into faunal degradation assessments, a limitation also encountered in this study, as we relied exclusively on ground-based camera trapping surveys. Given these differences, our indicators may need to be adjusted and tested in different hunting and biogeographical contexts. However, in Asian ecosystems, it is likely that the similarity with African forests in terms of body mass and biomass distribution of hunted mammal assemblages, where large terrestrial species such as elephants, wild cattle, and rhinoceros thrive^41^, may suggest a greater applicability of our indicators.

Beyond evaluating faunal degradation patterns, our assessment of species compositional shifts is useful in the spatial prioritization of conservation strategies and in monitoring and evaluating short- and long-term management and restoration efforts. We suggest that the continued use of our faunal degradation metric, derived from comparisons with reference areas, is necessary for comparison with faunas at hunted sites. Defining reference areas is pivotal and represents a target for restoration and defaunation mitigation projects and policies^10^. In many central African regions, information on undisturbed sites is increasingly available (Supplementary Table 5), but are often nonstandardized and may even be unavailable due to the lack of a regional data sharing initiative and platform. Given the wide-reaching impacts of ongoing environmental shifts^42^, additional reference (fewer disturbed) locations must be chosen and continuously monitored. Systems involving camera trap grids or line transects can be implemented by researchers, who generate standardized reference data for each zooregion^43^.

By coupling the indicators we advocate for, utilizing hunters’ reports of wildlife harvests enables a more comprehensive understanding of faunal degradation trajectories within hunting territories. For example, an upsurge in the prevalence of rodents in hunting records, a reduction in the average body mass of harvested animals, or a decrease in hunting yield may serve as early warning signs of increased defaunation in the ecosystem. This, in turn, can lead to the formulation of practical management recommendations, such as the establishment of no-take zones or collaborative efforts to curtail the harvest levels of specific species, with decisions ratified through deliberations within hunting associations and other relevant authorities.

The question of hunting sustainability falls beyond the scope of this study and remains a complex and unresolved concern. This is primarily due to a paucity of biological data for hunted species in tropical regions, particularly regarding their reproductive performance, which is fundamental for establishing harvest levels^44^. Furthermore, elucidating the dynamics of hunter-prey selection along degradation gradients, as underpinned by theoretical optimal foraging models^45,46^, is difficult. Ultimately, any long-term strategy for ensuring sustainable hunting hinges upon balancing the growth rate of a hunted species and its exploitation rate^8^. Nevertheless, the exact determination of the ecosystem’s carrying capacity required to support such maximal growth and harvest rates has not been determined. Our study offers some indirect insights into this overarching issue since we show that the CPUE indicators display a somewhat reverse U-shaped relationship with faunal degradation estimates. This observation is consistent with the concept of maximum sustainable yield^8^ and illustrates that hunting areas with intermediate degradation levels have high rates of biomass extraction per unit time or distance, surpassing those observed in hunting systems closer to reference conditions or more severely degraded.

To maintain community-driven initiatives effectively, it is essential to do more than just develop easily understandable, robust and ecologically relevant indicators. It requires ongoing dialogue and active participation to address the concerns and expectations of all stakeholders. It also involves implementing innovative natural resource management policies capable of mitigating potential economic downturns resulting from changes in hunting practices, as well as establishing legal frameworks that acknowledge and safeguard the status of resource users. Internal self-monitoring of management measures by local communities also remains very challenging, as traditional governance systems are scarce, making the sustainable and equitable governance of commons such as wildlife still very febrile^47^. As the world strives to expand protected areas to encompass 30% of the land area by 2030^48^, there is a pressing need to make local communities actively participate in innovative management models of natural resources^49^.

Our proposed approach integrates science-led assessments of intact faunas with community-based data collection in hunted areas, aligning seamlessly with the goal of expanding conservation efforts beyond strictly protected spaces. By establishing community-use zones and safeguarding landscapes, it is possible to preserve large-scale ecological connectivity while meeting the essential needs of human communities heavily dependent on natural resources for food and economic security.

## Methods

### Study area

The study was conducted within the wild meat supply area of Lastoursville (Fig. 1), the main town (approximately 12,000 inhabitants) in the Mulundu Department, eastern Gabon (28,000 inhabitants, and approximately 13,650 km² ^50,51^). Most of the resident human population is rural and is largely located along major roads, railway lines, and the Ogooué River, leaving the remaining forest areas sparsely inhabited.

Most of the landscape is covered by tropical evergreen forests^52,53^. The climate is typically equatorial, with 1,700 mm of annual rainfall distributed in two rainy and two dry seasons. The average temperature is consistently approximately 24.4 °C^54^.

Most forested areas are allocated for timber production, playing a significant role in the regional economy. Ivindo National Park (NP) lies along the northwestern boundary of the department (Fig. 1). The park was established in 2002 and serves as a dedicated area for the protection of biodiversity and habitats in the region. The presence of diverse and abundant fauna, including iconic species such as forest elephant (*Loxodonta cyclotis*) and leopard (*Panthera pardus*), suggests that a relatively intact ecosystem is maintained within park boundaries^55–58^.

### Hunting offtake monitoring

As part of the Sustainable Wildlife Management (SWM) Programme (https://www.swm-programme.info/), a baseline analysis, including socioeconomic surveys, was first carried out in 38 of the 40 groups of villages within the Mulundu Department. In March 2019, hunting offtake surveys started in three communities (Bembicani, Doumé, and Ndambi), and seven others (Kessipoughou, Malende, Nzondet, Bakoussou-Ndekabalandji, Lipaka 2, Baposso, Boundzi) gradually volunteered over the following years to form part of the SWM Programme and our study (Fig. 1).

The data collection team was composed of volunteer hunters, community surveyors and project supervisors. In each community, the hunters voluntarily engaged in hunting and offtake monitoring in accordance with the free, prior and informed consent (FPIC) principle.

To ensure anonymity, each participant in the monitoring program was assigned a unique identification number. Although the hunters did not receive monetary compensation, they were provided with incentives such as gifts or rewards once or twice a year to motivate their participation.

Community surveyors, who were residents of the villages, were selected regardless of whether they were hunters or not and were trained to monitor hunting activities. The number of community surveyors in each community ranged from one to three, depending on the number of active hunters. These surveyors were compensated monthly for their efforts.

Supervision of the monitoring process was provided by two technicians who were recruited and financially supported by the SWM Programme. They collected weekly data from the community surveyors in the villages. To ensure data quality and prevent potential fabrication, these supervisory visits were carried out randomly, avoiding a fixed verification schedule.

The wildlife offtake monitoring process involved equipping hunters with GPS devices, specifically the Garmin eTrex® 10, before their hunting expeditions. These devices were programmed to record location data at 30-second intervals and were activated by the hunters at the beginning of their hunting trips. GPS technology facilitated the documentation of hunting movements and spatial coverage, as well as the timeline and specific locations of catches.

Paper forms were used to collect various essential variables upon the hunters’ return from their expeditions. These variables included the hunter’s and GPS identification numbers, departure and return dates and times for the hunting trip, the method used for hunting (*e.g*., gun, snare, handpicked), details about the offtake (such as species, sex, age class, weight, and date and time of the kill), and the intended use of the harvested animals (self-consumption, sale, etc.). If the animal had been butchered, each section was individually weighed. In some cases, hunters were able to complete the forms themselves after their hunting trip, and in such instances, community surveyors meticulously verified the accuracy of the entered data.

To streamline the recording and processing of hunting and offtake information, a character recognition system coupled with a database was used. The recorded data then underwent systematic checks using predefined control rules and a dedicated interface. This data processing was semiautomated and implemented using R coding, resulting in the generation of a comprehensive set of indicators. These indicators were presented to the hunters in the form of dashboards, which were distributed to them every quarter, providing valuable insights into their hunting activities and their ecological impact.

The monitoring of hunting trips and offtake was initiated in March 2019 for the initial three partner communities. For this study, we restricted our analysis to monitoring data collected up to mid-September 2022, allowing some partner hunter communities to have up to 42 months of monitoring.

### Camera trapping survey

Along with offtake monitoring, camera trap (CT) surveys were also conducted to characterize the assemblages of ground-dwelling species within the hunting territories of each community. The CTs were deployed with a systematic grid layout, one CT every two km², that covered as much of each community’s hunting territory possible. Additionally, two “less hunted” sites were surveyed utilizing the same grid pattern. These sites included (1) Ivindo National Park (NP), with two CT grids installed in the southern and eastern parts, which we considered our reference site, and (2) a road-inaccessible sector of a logging concession granted to the FSC-certified *Precious Woods Gabon*-*Compagnie Equatoriale des Bois* (PWG-CEB), which is close to the Ivindo NP buffer area and was logged over a decade ago and surrounded by rivers (Fig. 1).

The CT surveys lasted from mid-June 2021 to mid-July 2022, except for the Ivindo NP survey, which was administered between April and June 2019. Throughout this timeframe, a total of 404 CTs (Bolyguard SG 2060X, Boly, Victoriaville, QC, Canada) were deployed within the forest for a minimum of one month. The CT devices were positioned at a height of 30–50 cm and were oriented to face small wildlife trails or trail crossings, drawing from previous research in the region^25^. Some minimal clearing of forest undergrowth was conducted to reduce false triggers while preserving the integrity of the CT setup site. The CT images were configured to capture five-second videos with the shortest possible trigger delay (0.8 s). The videos captured by the CT were analyzed using the open-access Timelapse Image Analysis system^59^.

For analysis purposes, all terrestrial and semiterrestrial species potentially subject to hunting were considered, except for the mandrill (*Mandrillus sphinx*), whose geographical distribution partially overlapped our study area (*i.e*., south of the Ogooué River^60^). Species classification followed the IUCN Red List of Threatened Species^61^. The species that were most difficult to identify were grouped into four species complexes^25^ (*e.g*., large-spotted genets, mongooses, forest squirrels, and small pangolins). Successive videos of the same species or species complex were considered an independent event if they were separated by at least 30 minutes^62^. The number of individuals was counted on each video. The maximum number of individuals observed within a single detection event was used to calculate the average number of individuals per species and per site studied.

### Assessing faunal degradation

From the camera trap (CT) data, we derived the total number of species observed and the estimated species richness at 1000 camera days using rarefaction and extrapolation curves. We also computed the proportions of rodents, ungulates, carnivores, apes and birds detected via CT as well as the mean body mass (MBM) of all the detection events (using reference biomass^63^) for all the surveyed areas. We additionally computed a dual index (BlueDuiker%) representing the percentage of blue duikers against all the other duikers (*Cephalophus spp*. and *Philantomba monticola*) detected. This index is adapted from earlier work^16,20,64^ but considers all duiker species rather than the more restrictive category of ‘red duiker’ since all medium- and large duiker species are likely to be less resistant to greater hunting pressure.

To assess the degree of faunal degradation in the surveyed hunting territories, we calculated the compositional similarity of the assemblage detected by each individual camera trap in the hunting territories (*n* = 301 CTs) with that detected by each individual camera trap in our reference area, the Ivindo NP (*n* = 38 CTs), for a total of 11,438 pairwise dissimilarity values (301 × 38). To do so, we used the Bray‒Curtis (BC) index^65^, which relies on species abundance (*N*):

1 $${BC}\text{i},\text{j}=\frac{{\sum }_{k}^{S}\left|{N}_{k,i}-{N}_{k,j}\right|}{{\sum }_{k}^{S}\left({N}_{k,i}+{N}_{k,j}\right)}$$

where *i* and *j* represent one assemblage detected by a CT within a hunting territory and within the Ivindo NP, respectively, and *k* varies from 1 to *S*, the overall species richness of the two assemblages. Here, the abundance of the *k*^th^ species at CT_*i*_ (*N*_*k,i*_) corresponds to the species daily detection rate (the ratio of the number of independent events for species *k* at CT_*i*_ to the number of functioning days at CT_*i*_), weighted by the average number of individuals per site for that species to account for the gregarious nature of certain species. The BC index ranges from 0 (indicative of a composition entirely resembling that of the Ivindo NP) to 1 (indicative of an entirely distinct composition) and is hereafter referred to as the degree of faunal degradation.

Among the many coefficients used by ecologists for the analysis of assemblage data, the BC index is one of the most popular measures of multivariate dissimilarity in community ecology^65^. Because such analyses are sensitive to extremely rare species and species-poor assemblages, a final selection criterion was to consider only those species detected by at least three CTs and CTs that detected at least 3 species, as previously discussed^25,43^. The median dissimilarity between all pairs of CTs from each community and the Ivindo NP was subsequently used to locate all hunting territories along a faunal degradation gradient.

### Characterizing hunting practices and offtake

For each hunter community studied, we described the total number of hunters who had declared any harvest, the number of active hunters (on average, more than five hunting trips recorded per year; see Supplementary Fig. 3 to visualize the recurrence of hunting activity for the hunters monitored), the period monitored, the proportion of each type of hunt (strict gun hunt, strict trap hunt, or mixed hunt), the species richness observed and estimated based on 500 recorded hunting trips, and the total biomass extracted per year by all hunters we monitored.

To determine the spatial extent of hunting activities, we computed the utilization distribution (UD) based on GPS hunter follow data using a movement-based kernel density estimation^66,67^. This method improves the spatial resolution of UD estimates by considering activity times between serially correlated relocations rather than simply considering the spatial density of these relocations as if they were unlinked. We computed UDs (up to the 0.95 isopleth) for all individual hunters who were GPS-tracked at least once (*n* = 234 hunters and 5,595 hunting trips) and stacked all UDs to determine the total area exploited by hunters from the same community.

We then calculated 33 indicators of hunting offtake and pressure for each surveyed community via participatory monitoring. These indicators include the following information: (1) hunting method, proportion of individuals gunshot (#1) or caught in wire snares (#2), proportion of individuals caught at night (#3) and during the day (#4), mean duration (#5) and distance (#6) and proportion of night-time activity (#7) of gun hunting trips; (2) catch composition, proportion of rodents (#8), ungulates (#9) primates (#10) and birds (#11) in hunting catches, BlueDuiker% based on harvested duikers (#12), mean body mass of hunter offtake (#13); (3) hunter returns, percentage of unsuccessful hunting trips (#14), strict gun hunting trips (#15) and strict trap hunting trips (#16), annual exploitation rate considering all species expressed in kg/km².y (#17), mean biomass harvested per hunting trip, per hour and per kilometer considering all species (#18-20) and considering only rodents (#21-23), ungulates (#24-26), primates (#27-29) and birds (#30-32); and (4) wild meat use, proportion of wild meat pieces traded (#33). Based on a comprehensive collection of prior research as part of the WILDMEAT initiative (https://wildmeat.org/) and toolkit (https://www.wildmeat.org/toolkit/indicators/) and our empirical knowledge of hunting systems in the study area combined with finer-grained monitoring methods, we considered this suite of indicators as potential predictors of faunal degradation in hunted wildlife assemblages (see Supplementary Table 4 for hypotheses and expected trends).

### Correlation of hunting indicators with the faunal degradation degree

We first tested pairwise Pearson correlations between all hunting offtake and pressure indicators to better understand the dynamics of the studied hunting systems (Supplementary Fig. 5). Then, we tested for significant correlations between all 33 indicators and the degree of faunal degradation in the community hunting territory (using the median value for each community of the BC dissimilarities with the reference) (Supplementary Table 4). A Bonferroni adjustment of *P* values was made because of the multiple comparisons.

All analyses were performed in R software using the “adehabitatHR” package^68^ to estimate hunter space use, “iNEXT”^69^ for standardized species richness in camera trap and offtake data, “vegan”^70^ for dissimilarity-based analyses and “Hmisc”^71^ for pairwise correlations.

### Supplementary Information

Supplementary Information

## Data Availability

Correspondence and requests for materials should be addressed to Davy Fonteyn. All raw data are freely available on the Dataverse depository (10.18167/DVN1/75ZDAM, 10.18167/DVN1/TNBXCN).

## References

[1] Terborgh, J. et al. Ecological meltdown in predator-free forest fragments. *Science* **294**, 1923–1926 (2001). 11729317 10.1126/science.1064397

[2] Lacher, T. E. Jr. et al. The functional roles of mammals in ecosystems. *J. Mammal.* **100**, 942–964 (2019). 10.1093/jmammal/gyy183

[3] Fa, J. E., Peres, C. A. & Meeuwig, J. Bushmeat exploitation in tropical forests: an intercontinental comparison. *Conserv. Biol.* **16**, 232–237 (2002). 35701970 10.1046/j.1523-1739.2002.00275.x

[4] Benítez-López, A. et al. The impact of hunting on tropical mammal and bird populations. *Science* **356**, 180–183 (2017). 28408600 10.1126/science.aaj1891

[5] Ripple, W. J. et al. Bushmeat hunting and extinction risk to the world’s mammals. *R. Soc. Open Sci.* **3**, 160498 (2016). 27853564 10.1098/rsos.160498 PMC5098989

[6] Peres, C. A. & Dolman, P. M. Density compensation in neotropical primate communities: evidence from 56 hunted and nonhunted Amazonian forests of varying productivity. *Oecologia* **122**, 175–189 (2000). 28308371 10.1007/PL00008845

[7] Peres, C. A., Emilio, T., Schietti, J., Desmoulière, S. J. M. & Levi, T. Dispersal limitation induces long-term biomass collapse in overhunted Amazonian forests. *Proc. Natl Acad. Sci.* **113**, 892–897 (2016). 26811455 10.1073/pnas.1516525113 PMC4743805

[8] Fa, J. E., Funk, S. M. & Nasi, R. *Hunting Wildlife in the Tropics and Subtropics*. (Cambridge University Press, 2022).

[9] Giacomini, H. C. & Galetti, M. An index for defaunation. *Biol. Conserv.* **163**, 33–41 (2013). 10.1016/j.biocon.2013.04.007

[10] Poulsen, J. R., Maicher, V., Malinowski, H. & DeSisto, C. Situating defaunation in an operational framework to advance biodiversity conservation. *BioScience* **73**, 721–727 (2023). 37854893 10.1093/biosci/biad079 PMC10580966

[11] Coad, L. et al. *Towards a Sustainable, Participatory and Inclusive Wild Meat Sector*. (Bogor, Indonesia: CIFOR, 2019).

[12] Froese, G. Z. L. et al. Coupling paraecology and hunter GPS self-follows to quantify village bushmeat hunting dynamics across the landscape scale. *Afr. J. Ecol.* **60**, 229–249 (2022). 10.1111/aje.12956

[13] Riddell, M. et al. Combining offtake and participatory data to assess the sustainability of a hunting system in northern Congo. *Afr. J. Ecol.* **60**, 250–267 (2022). 10.1111/aje.13001

[14] Ingram, D. J. et al. Indicators for wild animal offtake: methods and case study for African mammals and birds. *Ecol. Soc.* **20**, (2015).

[15] McNamara, J. et al. Long-term spatio-temporal changes in a West African bushmeat trade system. *Conserv. Biol.* **29**, 1446–1457 (2015). 26104770 10.1111/cobi.12545 PMC4745032

[16] Marrocoli, S. et al. Using wildlife indicators to facilitate wildlife monitoring in hunter-self monitoring schemes. *Ecol. Indic.* **105**, 254–263 (2019). 10.1016/j.ecolind.2019.05.050

[17] Fa, J. E. et al. Correlates of bushmeat in markets and depletion of wildlife. *Conserv. Biol.* **29**, 805–815 (2015). 25580729 10.1111/cobi.12441

[18] Grantham, H. S. et al. Spatial priorities for conserving the most intact biodiverse forests within Central Africa. *Environ. Res. Lett.* **15**, 0940b5 (2020). 10.1088/1748-9326/ab9fae

[19] Vancutsem, C. et al. Long-term (1990–2019) monitoring of forest cover changes in the humid tropics. *Sci. Adv.* **7**, eabe1603 (2021). 33674308 10.1126/sciadv.abe1603 PMC7935368

[20] Hongo, S. et al. Predicting bushmeat biomass from species composition captured by camera traps: Implications for locally based wildlife monitoring. *J. Appl. Ecol.* **59**, 2567–2580 (2022). 10.1111/1365-2664.14257

[21] Ávila Martin, E. et al. Wild meat hunting and use by sedentarised Baka Pygmies in southeastern Cameroon. *PeerJ* **8**, e9906 (2020). 32995086 10.7717/peerj.9906 PMC7502248

[22] Robinson, J. G. & Bennett, E. L. Having your wildlife and eating it too: an analysis of hunting sustainability across tropical ecosystems. *Anim. Conserv. forum* **7**, 397–408 (2004). 10.1017/S1367943004001532

[23] Rist, J., Milner-Gulland, E. J., Cowlishaw, G. & Rowcliffe, M. Hunter reporting of catch per unit effort as a monitoring tool in a bushmeat-harvesting system. *Conserv. Biol.* **24**, 489–499 (2010). 20491849 10.1111/j.1523-1739.2010.01470.x

[24] Lhoest, S. et al. Conservation value of tropical forests: distance to human settlements matters more than management in Central Africa. *Biol. Conserv.* **241**, 108351 (2020). 10.1016/j.biocon.2019.108351

[25] Fonteyn, D. et al. Wildlife trail or systematic? Camera trap placement has little effect on estimates of mammal diversity in a tropical forest in Gabon. *Remote Sens. Ecol. Conserv.* **7**, 321–336 (2021). 10.1002/rse2.191

[26] Verbeeck, H., Boeckx, P. & Steppe, K. Tropical forests: Include Congo basin. *Nature* **479**, 179–179 (2011). 22071754 10.1038/479179b

[27] Fonteyn, D. et al. FauneFAC: boite à outils méthodologique pour la mise en place d’inventaires par pièges photographiques. https://www.gembloux.ulg.ac.be/faunefac/ (2021).

[28] Zwerts, J. A. et al. Methods for wildlife monitoring in tropical forests: Comparing human observations, camera traps, and passive acoustic sensors. *Conserv. Sci. Pract.* **3**, e568 (2021). 10.1111/csp2.568

[29] Haurez, B. et al. *Elaboration et mise en oeuvre d’un plan de gestion de la faune. Guide Technique à destination des gestionnaires des forêts de production d’Afrique centrale*. (Presses Agronomiques de Gembloux, 2020).

[30] Janzen, D. H., Hallwachs, W., Jimenez, J. & Gámez, R. The role of the parataxonomists, inventory managers and taxonomists in Costa Rica’s nationalbiodiversity inventory. In *Biodiversity Prospecting* (eds Reid, W. V. et al.) 223–254 (World Resources Institute, Washington, D. C., 1993).

[31] El Bizri, H. R. et al. Involving local communities for effective citizen science: determining game species’ reproductive status to assess hunting effects in tropical forests. *J. Appl. Ecol.* **58**, 224–235 (2021). 10.1111/1365-2664.13633

[32] Mayor, P., El Bizri, H., Bodmer, R. E. & Bowler, M. Assessment of mammal reproduction for hunting sustainability through community-based sampling of species in the wild. *Conserv. Biol.* **31**, 912–923 (2017). 27917537 10.1111/cobi.12870

[33] Ziegler, S. et al. Mapping bushmeat hunting pressure in Central Africa. *Biotropica* **48**, 405–412 (2016). 10.1111/btp.12286

[34] Benítez-López, A., Santini, L., Schipper, A. M., Busana, M. & Huijbregts, M. A. Intact but empty forests? Patterns of hunting-induced mammal defaunation in the tropics. *PLoS Biol.* **17**, e3000247 (2019). 31086365 10.1371/journal.pbio.3000247 PMC6516652

[35] Alves-Pinto, H. et al. Opportunities and challenges of other effective area-based conservation measures (OECMs) for biodiversity conservation. *Perspect. Ecol. Conserv.* **19**, 115–120 (2021).

[36] Cole, L. C. The population consequences of life history phenomena. *Q. Rev. Biol.* **29**, 103–137 (1954). 13177850 10.1086/400074

[37] Fa, J. E. & Purvis, A. Body size, diet and population density in Afrotropical forest mammals: a comparison with neotropical species. *J. Anim. Ecol.* **66**, 98–112 (1997). 10.2307/5968

[38] Peters, R. H. *The Ecological Implications of Body Size*. (Cambridge University Press, 1983).

[39] Fa, J. E. & Peres, C. A. Game vertebrate extraction in African and Neotropical forests: An intercontinental comparison. in *Conservation of Exploited Species* (eds. Reynolds, J. D., Mace, G. M., Redford, K. H. & Robinson, J. G.) 203–241 (Cambridge University Press, 2001).

[40] Peres, C. A. Effects of hunting on western Amazonian primate communities. *Biol. Conserv.* **54**, 47–59 (1990). 10.1016/0006-3207(90)90041-M

[41] Corlett, R. T. The impact of hunting on the mammalian fauna of tropical Asian forests. *Biotropica* **39**, 292–303 (2007). 10.1111/j.1744-7429.2007.00271.x

[42] Bush, E. R. et al. Long-term collapse in fruit availability threatens Central African forest megafauna. *Science* **370**, 1219–1222 (2020). 32972990 10.1126/science.abc7791

[43] Fonteyn, D. et al. Biogeography of central African forests: determinants, ongoing threats and conservation priorities of mammal assemblages. *Diversity Distrib.* **29**, 698–712 (2023). 10.1111/ddi.13677

[44] Van Vliet, N. & Nasi, R. What do we know about the life-history traits of widely hunted tropical mammals? *Oryx* **53**, 670–676 (2019). 10.1017/S0030605317001545

[45] Griffiths, B. M. et al. Revisiting optimal foraging theory (OFT) in a Changing Amazon: implications for conservation and management. *Hum. Ecol.* **50**, 545–558 (2022). 10.1007/s10745-022-00320-w

[46] Alvard, M. Indigenous hunting in the Neotropics: conservation or optimal foraging? in *Behavioral Ecology and Conservation Biology* (ed. Caro, T.) (Oxford University Press, New York, Oxford, 1998).

[47] Fargeot, C., Drouet-Hoguet, N., Le Bel, S. & Billand, A. Evidence of social regulation in access of wildlife in the Village Hunting Territory, the case of the Central African Republic. in (XIV WORLD FORESTRY CONGRESS, Durban, South Africa, 2015).

[48] CBD. *The Kunming-Montreal Global Biodiversity Framework*. https://www.cbd.int/doc/c/e6d3/cd1d/daf663719a03902a9b116c34/cop-15-l-25-en.pdf (2022).

[49] Vermeulen, C., Julve, C., Doucet, J.-L. & Monticelli, D. Community hunting in logging concessions: towards a management model for Cameroon’s dense forests. *Biodivers. Conserv* **18**, 2705–2718 (2009). 10.1007/s10531-009-9614-6

[50] MEFEPA & WRI. MEFEPA et WRI. 2017. Atlas forestier de la République du Gabon, gab.forest-atlas.org (page web consultée le 24 novembre 2020) (2017).

[51] Direction Générale de la Statistique, Résultats globaux du Recensement Général de la Population et des Logements de 2013 du Gabon (RGPL-2013). (2015).

[52] Fayolle, A. et al. Patterns of tree species composition across tropical African forests. *J. Biogeogr.* **41**, 2320–2331 (2014). 10.1111/jbi.12382

[53] Réjou-Méchain, M. et al. Unveiling African rainforest composition and vulnerability to global change. *Nature* **593**, 90–94 (2021). 33883743 10.1038/s41586-021-03483-6

[54] Fick, S. E. & Hijmans, R. J. WorldClim 2: new 1-km spatial resolution climate surfaces for global land areas. *Int. J. Climatol.* **37**, 4302–4315 (2017). 10.1002/joc.5086

[55] Rosin, C. & Poulsen, J. R. Hunting-induced defaunation drives increased seed predation and decreased seedling establishment of commercially important tree species in an Afrotropical forest. *For. Ecol. Manag.* **382**, 206–213 (2016). 10.1016/j.foreco.2016.10.016

[56] Terborgh, J. et al. Megafaunal influences on tree recruitment in African equatorial forests. *Ecography* **39**, 180–186 (2016). 10.1111/ecog.01641

[57] Laguardia, A. et al. Nationwide abundance and distribution of African forest elephants across Gabon using non-invasive SNP genotyping. *Glob. Ecol. Conserv.* **32**, e01894 (2021).

[58] Bahaa-el-din, L. et al. Effects of human land-use on Africa’s only forest-dependent felid: The African golden cat Caracal aurata. *Biol. Conserv.* **199**, 1–9 (2016). 10.1016/j.biocon.2016.04.013

[59] Greenberg, S. The Timelapse user guide version 2.2.5.0. (2022).

[60] Fonteyn, D. et al. Range extension of the agile mangabey (Cercocebus agilis) and of the mandrill (Mandrillus sphinx) in eastern Gabon evidenced by camera traps. *Afr. J. Ecol.* **60**, 1267–1270 (2022). 10.1111/aje.13061

[61] IUCN. The IUCN Red List of Threatened Species. Version 2022-2. https://www.iucnredlist.org (2022).

[62] Meek, P. D. et al. Recommended guiding principles for reporting on camera trapping research. *Biodivers. Conserv.* **23**, 2321–2343 (2014). 10.1007/s10531-014-0712-8

[63] Kingdon, J. et al. *Mammals of Africa (6 Vols)*. (London, 2013).

[64] Yasuoka, H. et al. Changes in the composition of hunting catches in southeastern Cameroon: a promising approach for collaborative wildlife management between ecologists and local hunters. *Ecol. Soc.* **20**, 25 (2015). 10.5751/ES-08041-200425

[65] Legendre, P. & Legendre, L. F. J. *Numerical Ecology*. (Elsevier Science, 1998).

[66] Benhamou, S. Dynamic approach to space and habitat use based on biased random bridges. *PLoS ONE* **6**, e14592 (2011). 21297869 10.1371/journal.pone.0014592 PMC3027622

[67] Benhamou, S. & Cornélis, D. Incorporating movement behavior and barriers to improve kernel home range space use estimates. *J. Wildl. Manag.* **74**, 1353–1360 (2010).

[68] Calenge, C. The package “adehabitat” for the R software: A tool for the analysis of space and habitat use by animals. *Ecol. Model.* **197**, 516–519 (2006). 10.1016/j.ecolmodel.2006.03.017

[69] Hsieh, T. C., Ma, K. H. & Chao, A. iNEXT: iNterpolation and EXTrapolation for species diversity. R package version 2.0. 12. (2016).

[70] Oksanen, J. et al. Vegan: Community ecology package. (2019).

[71] Harrel Jr, F. Hmisc: Harrell Miscellaneous, R package version 5.1-0. (2023).

