# Supplementary material for: Hunting indicators for community-led wildlife management in tropical Africa
Source: NPJ Biodivers. 2024 Jul 18;3:15. doi: 10.1038/s44185-024-00048-4 (PMC11332190; doi:10.1038/s44185-024-00048-4)
Supplement: Supplementary file 1 — Supplementary Information [file 44185_2024_48_MOESM1_ESM.pdf]

Supplementary Information : Hunting indicators for community-led wildlife management in tropical Africa

Davy Fonteyn[1,2,3]*, Adeline Fayolle[3], Julia E. Fa[4,5], Hadrien Vanthomme[1,2], Philippe Vigneron[1,2], Cédric Vermeulen[3], Rémi Malignat[1,2], Benoît Konradowski[1,2], Mexan Noel Yia Okanabene[1,2], Stéphane Axel Dibotty-di Moutsing[1,2], Samuel Pereira Dias[1,2], Christophe Deniau[1,2], Guillaume Cornu[1,2], Marion Groschêne[1,2], Daniel Cornélis[1,2]

Affiliations:

[1]CIRAD, UPR Forêts et Sociétés, Campus International de Baillarguet, Montpellier Cedex 5, France.

[2]Forêts et Sociétés, Univ Montpellier, CIRAD, Montpellier, France.

[3] Université de Liège – Gembloux Agro-Bio Tech, FORIL, Unité Gestion des Ressources Forestières, Passage des Déportés 2, BE-5030 Gembloux, Belgium.

[4] Department of Natural Sciences, Faculty of Science and Engineering, Manchester Metropolitan University, Manchester, United Kingdom.

[5] Center for International Forestry Research (CIFOR), Kota Bogor, Jawa Barat, Indonesia.

***Corresponding author. Email: davy_fo@hotmail.com (D.F.)**

Supplementary Figure 1: **Terrestrial and semiterrestrial species assemblages detected by camera traps within the study area**. (**a**) The total number of species observed and the estimated species richness at 1,000 camera days using rarefaction and extrapolation curves; (**b**) the proportion of rodents ( ), medium-sized ( ) and large ( ) ungulate, blue duikers ( ), carnivores ( ), apes ( ), red river hogs ( ) and terrestrial birds ( ) in all camera trap events along the faunal degradation gradient; (**c**) the mean body mass of all camera trap events excluding elephant and great apes; and (**d**) the percentage of blue duikers against all duikers (*Cephalophus spp*. and *Philantomba monticola*) detected along the faunal degradation gradient. The curves and points are colored according to the median Bray–Curtis index in Fig. 2.

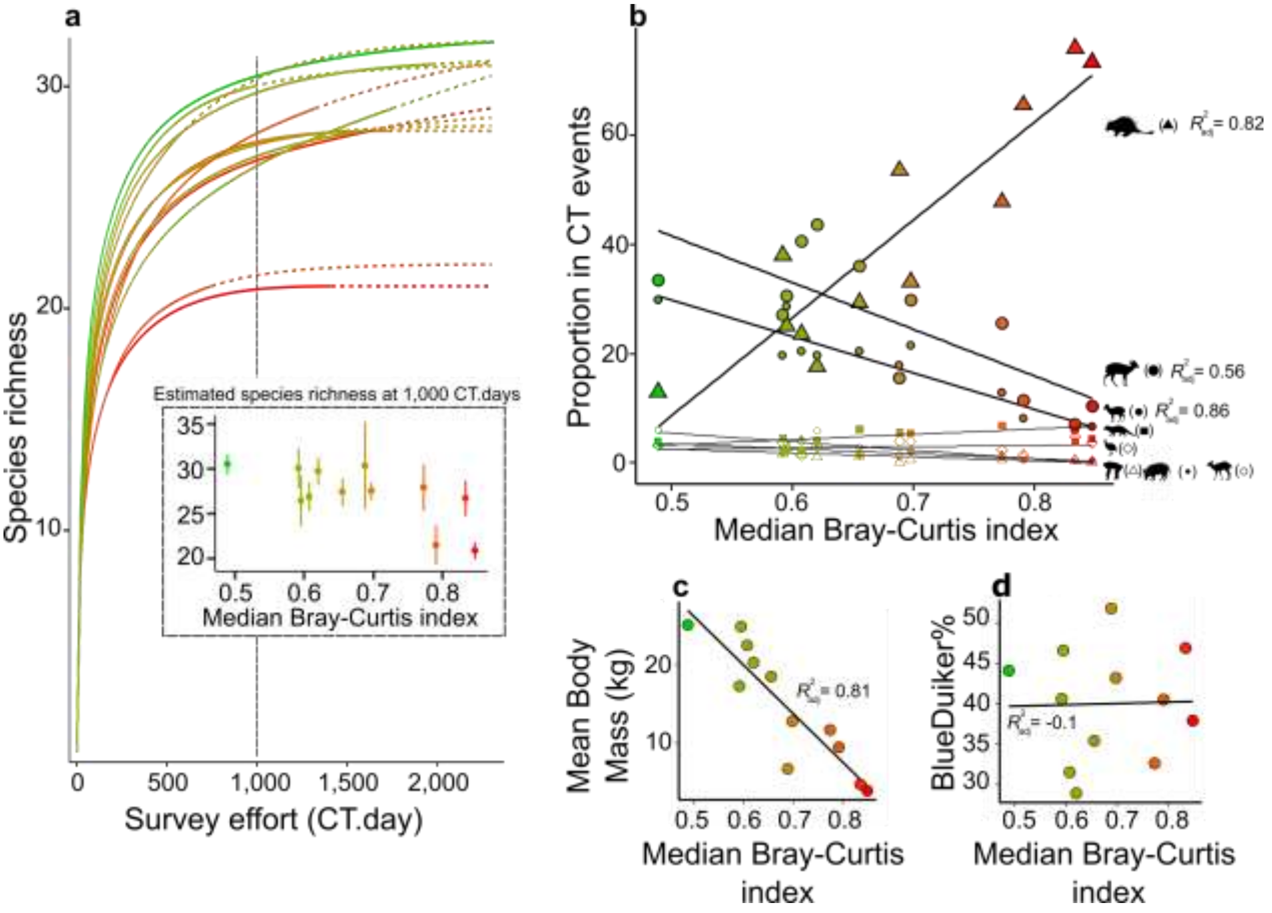

Supplementary Figure 2: **Species detection rates along the faunal degradation gradient ordered by species groups and decreasing biomass**. Species detection rates are expressed as the number of independent detection events per 100 camera days. The total number of independent events and reference biomass[63] are displayed for each species or species complex. Sites are organized in the bar charts by increasing faunal degradation (Extended Data Table 1).

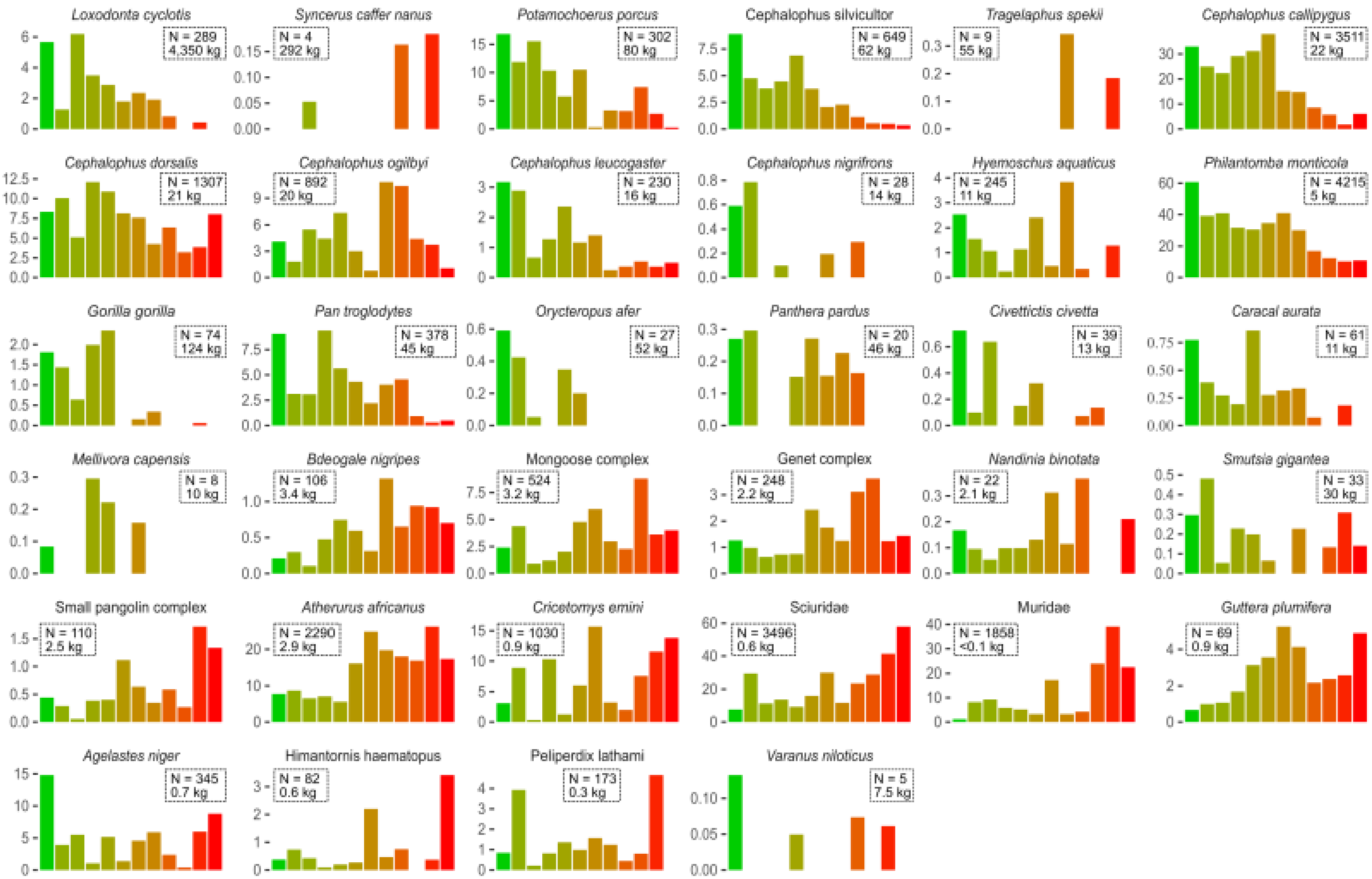

Supplementary Figure 3: **Hunter activity**. The frequency of hunting trips per year is displayed for every hunter who participated in the monitoring of hunting activities for at least 90 days (n = 239 hunters).

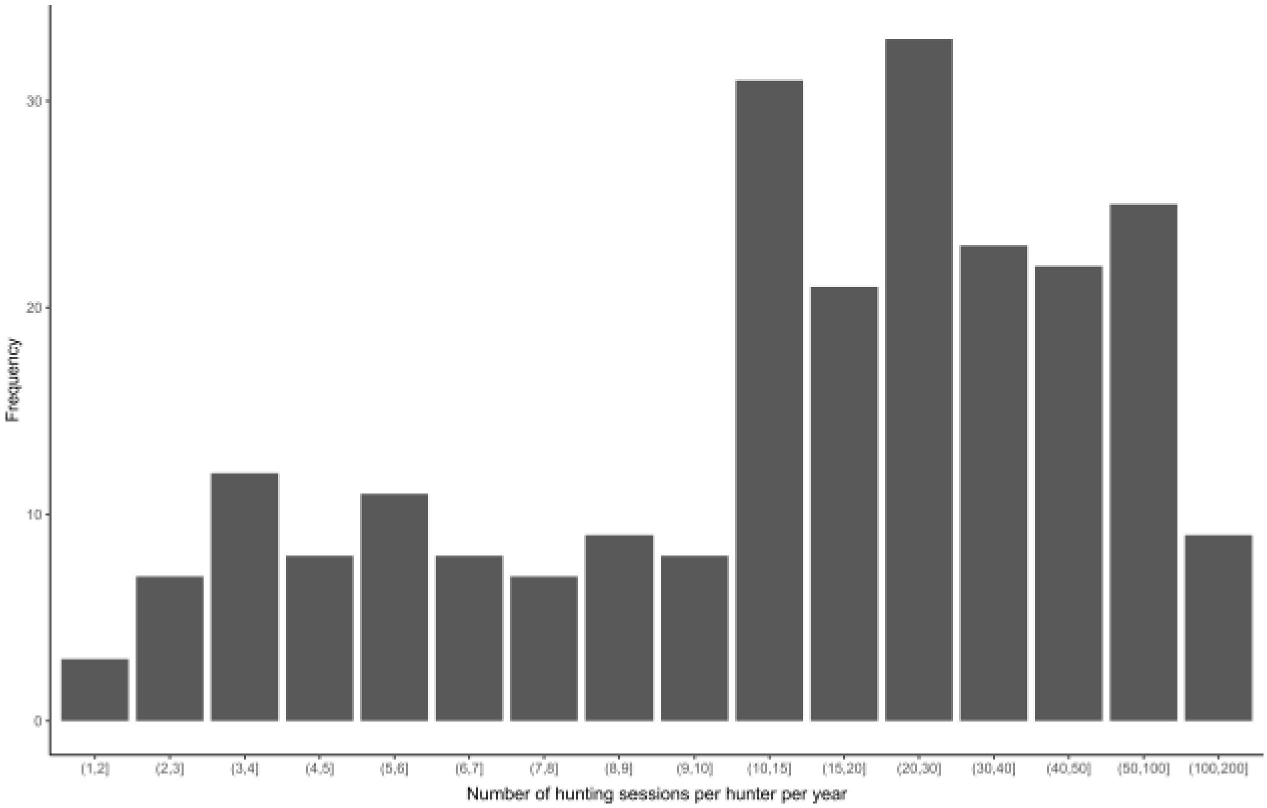

Supplementary Figure 4: **Offtake composition**. The total number of individuals killed and their respective contributions to the total offtake and biomass harvested are presented for the species responsible for 95% of the catches.

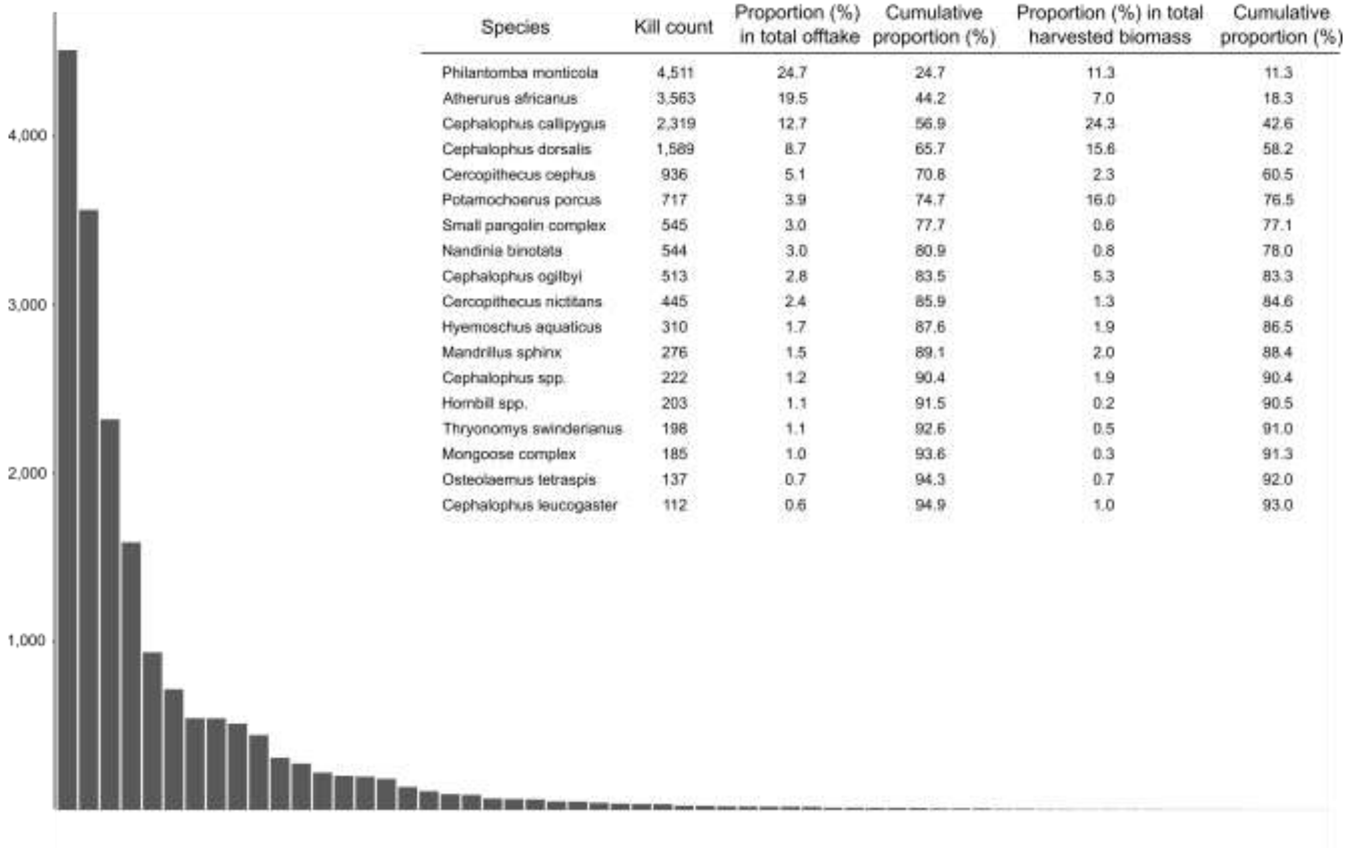

Species	Kill count	Proportion (%) in total offtake	Cumulative proportion (%)	Proportion (%) in total harvested biomass	Cumulative proportion (%)
Philantomba monticola	4,511	24.7	24.7	11.3	11.3
Atherurus africanus	3,563	19.5	44.2	7.0	18.3
Cephalophus callipygus	2,319	12.7	56.9	24.3	42.6
Cephalophus dorsalis	1,589	8.7	65.7	15.6	58.2
Cercopithecus cephus	936	5.1	70.8	2.3	60.5
Potamochoerus porcus	717	3.9	74.7	16.0	76.5
Small pangolin complex	545	3.0	77.7	0.6	77.1
Nandinia binotata	544	3.0	80.9	0.8	78.0
Cephalophus ogilbyi	513	2.8	83.5	5.3	83.3
Cercopithecus nictitans	445	2.4	85.9	1.3	84.6
Hyemoschus aquaticus	310	1.7	87.6	1.9	86.5
Mandrillus sphinx	276	1.5	89.1	2.0	88.4
Cephalophus spp.	222	1.2	90.4	1.9	90.4
Hornbill spp.	203	1.1	91.5	0.2	90.5
Thryonomys swinderianus	198	1.1	92.6	0.5	91.0
Mongoose complex	185	1.0	93.6	0.3	91.3
Osteolaemus tetraspis	137	0.7	94.3	0.7	92.0
Cephalophus leucogaster	112	0.6	94.9	1.0	93.0

Supplementary Figure 5: Correlogram showing correlations between hunting offtake indicators. Pairwise Pearson correlation coefficients for all pairs of indicators (with more intense colors indicating more extreme correlations) and correlations not significantly different from 0 are represented by a white box.

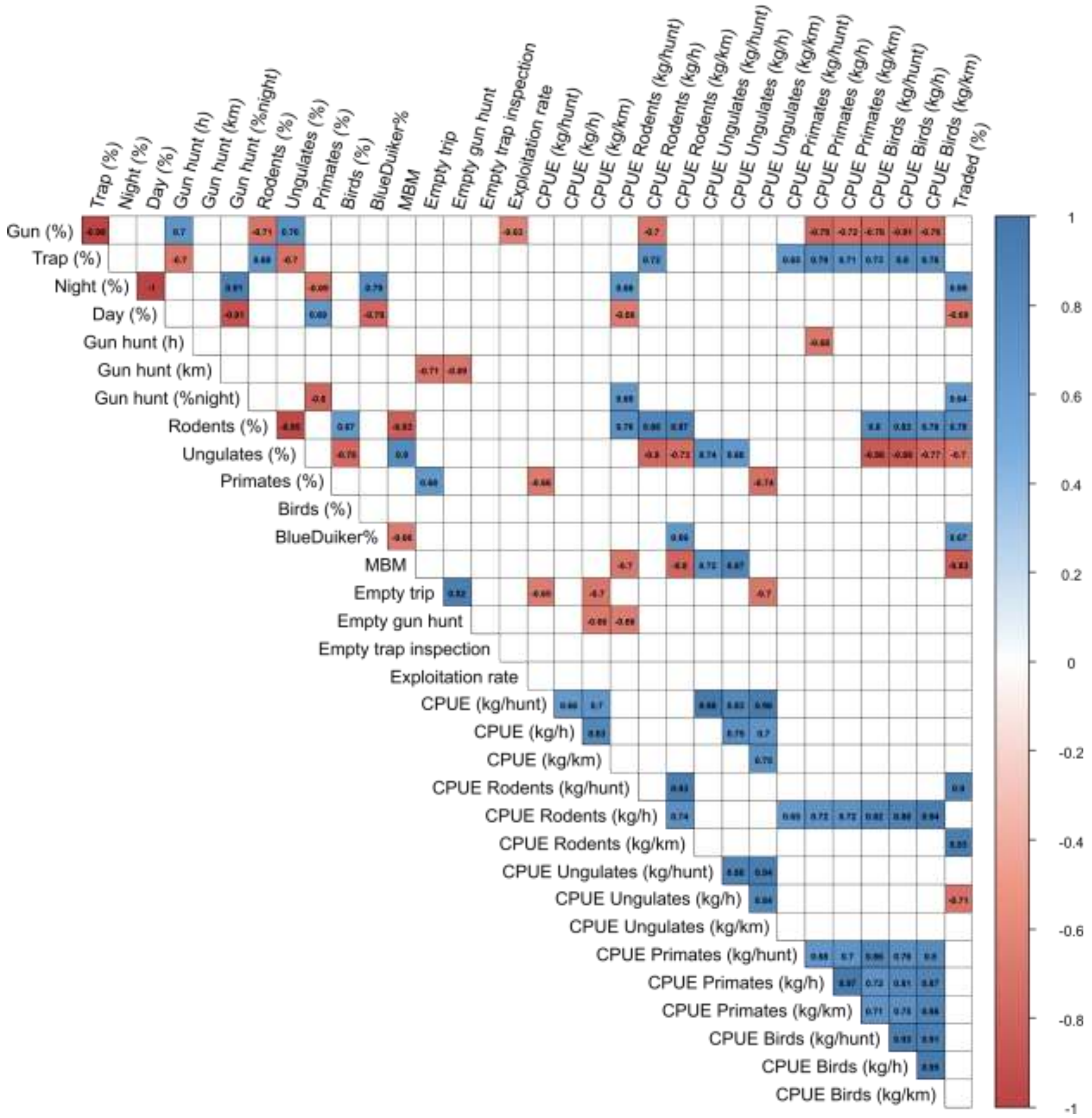

Arboreal primate hunting was associated with shorter hunting trips and a greater proportion of unsuccessful attempts, suggesting unique challenges in this aspect of hunting. Notably, there were striking similarities in hunting patterns between primate and bird hunting, hinting at shared strategies. The study also revealed that the blue-to-large duiker ratio increased with faunal degradation, mainly due to the greater number of blue duikers captured at night in these more degraded areas.

Supplementary Table 1: Diversity and composition of terrestrial and semiterrestrial faunal assemblages detected by camera traps (CT) in the study area.

	Ivindo National Park	Baposso	PWG-CEB	Nzondet	Ndambi	Doume	Bakoussou-Ndekabalandji	Boundzi	Malende	Kessipoughou	Lipaka2	Bembicani
**a) CT survey characteristics (N=374)**												
Number of CT	38	23	30	29	53	45	15	46	17	15	32	31
Survey effort (camera.days)	2,316	980	1,748	1,587	1,966	1,488	592	1,726	1,319	746	1,626	1,409
**b) Diversity and composition of wildlife**												
Species richness observed	32	30	29	28	31	28	28	28	29	21	28	21
Species richness estimated at 1000 camera.days & CI0.95	30.5 (29-32)	30.0 (28-32)	26.4 (24-29)	26.9 (25-28)	29.7 (28-31)	27.4 (26-29)	30.3 (25-35)	27.5 (27-29)	27.9 (25-30)	21.5 (19-24)	26.7 (25-29)	20.9 (20-22)
Percentage in CT detections (%)												
Rodents*	12.96	38.03	25.12	23.60	17.61	29.39	53.59	33.16	47.82	65.59	75.99	73.41
Ungulates	72.72	51.19	64.67	66.21	70.14	60.24	34.78	53.89	40.19	20.94	13.71	17.32
Blue duiker	29.76	19.68	28.56	20.37	19.69	20.34	17.80	21.41	12.79	8.06	5.77	6.51
Medium ungulates †	33.49	27.10	30.54	40.60	43.66	36.04	15.57	29.82	25.58	11.39	7.05	10.41
Large ungulates ‡	6.01	2.80	3.28	3.77	5.80	2.74	1.31	2.04	1.27	0.46	0.48	0.25
Red river hog	3.46	1.61	2.29	1.47	1.00	1.12	0.10	0.63	0.56	1.04	0.40	0.15
Carrnivores	3.88	4.48	2.40	2.15	4.07	6.06	5.46	5.38	6.75	11.28	3.89	4.34
Small carnivores §	3.11	3.99	2.24	1.91	3.07	5.67	5.06	4.96	6.59	11.28	3.77	4.34
Medium and Large carnivores ¶	0.77	0.49	0.16	0.24	1.00	0.39	0.40	0.42	0.16	/	0.12	/
Elephants	2.69	0.63	3.49	1.81	1.21	0.68	1.11	1.04	0.56	/	0.20	/
Apes	3.58	1.40	1.93	4.46	3.20	1.17	0.71	2.14	1.75	0.46	0.20	0.30
Pangolins	0.50	0.49	0.10	0.49	0.52	0.88	0.40	0.47	0.64	0.35	1.28	1.04
Small pangolins	0.30	0.21	0.05	0.29	0.35	0.83	0.40	0.26	0.64	0.23	1.08	0.94
Giant pangolin	0.21	0.28	0.05	0.20	0.17	0.05	/	0.21	/	0.12	0.20	0.10
Terrestrial birds #	3.22	3.57	2.24	1.27	2.90	1.42	3.94	3.92	2.22	1.38	4.69	3.60
Mean body mass of all CT detections (kg)	196	59	288	153	129	62	73	89	48	10	17	4
Mean body mass of all CT detections except elephants & apes (kg)	25	17	25	22	20	18	7	13	12	10	5	4

BlueDuiker%	44.1	40.6	46.6	31.5	28.9	35.4	51.8	43.2	32.6	40.5	46.9	37.9
**c) Median Bray–Curtis index**												
Median Bray–Curtis faunistic dissimilarity	0.49	0.59	0.60	0.61	0.62	0.66	0.69	0.70	0.77	0.79	0.83	0.85

* including *Atherurus africanus*, *Cricetomys emini* and all species from the Muridae and Sciuridae families
† including *Hyemoschus aquaticus* and all *Cephalophus* spp., except for *Cephalophus silvicultor*
‡ including *Cephalophus silvicultor*, *Syncerus caffer nanus* and *Tragelaphus spekii*
§ including *Bdeogale nigripes*, *Civettictis civetta*, *Nandinia binotata* and the Genet and Mongoose species complexes
¶ including *Mellivora capensis*, *Caracal aurata* and *Panthera pardus*
# *Agelastes niger*, *Guttera plumifera*, *Himantornis haematopus* and *Peliperdix lathami*

Supplementary Table 2: Characteristics of the 10 hunter communities surveyed on the basis of the results of the offtake survey and hunting activities.

	Baposso	Nzondet	Ndambi	Doume	Bakoussou-Ndekabalandji	Boundzi	Malende	Kessipoughou	Lipaka2	Bembicani
**Offtake survey characteristics**										
Number of participating hunters	18	28	46	25	43	25	15	29	38	68
Number of active hunters (>5 hunts.y^{-1})	9	13	16	15	18	11	9	13	25	47
Number of hunting sessions declared	510	443	1,220	1,857	724	425	879	976	977	4,959
Survey effort (days)	496	537	1,270	1,273	534	471	676	681	534	1,273
**Type of hunts**										
Strict gun hunt sessions (%)	82.2	91.8	94.0	92.9	65.5	53.8	29.3	75.6	79.6	45.1
Strict trap visit sessions (%)	4.0	7.5	4.3	5.7	27.6	37.4	56.7	11.3	13.7	48.4
Mixed hunt sessions (%)	13.6	0.7	1.5	1.0	6.9	8.8	12.6	13.0	4.5	6.0
**Spatial extent of hunting activities**										
Hunting territory size (MBK 95%) (km^2)	55	93	217	195	75	49	52	53	100	150
**Offtake diversity**										
Species richness	23	19	30	29	40	32	31	28	31	40
Species richness estimated at 500 hunting trips & CI0.95	22.9 (19-26)	19.6 (16-23)	23.9 (21-26)	21.8 (20-23)	36.9 (34-40)	32.6 (29-36)	28.8 (27-31)	24.0 (22-26)	27.9 (26-30)	27.8 (27-29)
**Annual harvested biomass**										
Total biomass extracted per year by the hunters (kg/year)	3,715	3,846	8,272	11,018	6,848	5,818	4,088	3,800	6,860	11,721

Supplementary Table 3: Hunting offtake and pressure indicators in the 10 hunter communities surveyed. CPUE stands for catch-per-unit-effort.

	Baposso	Nzondet	Ndambi	Doume	Bakoussou-Ndekabalandji	Boundzi	Malende	Kessipoughou	Lipaka2	Bembicani
**Hunting method**										
#1 Proportion of shot individuals	73.4	96.7	95.1	94.6	75.2	70.7	47.5	73.8	82.0	64.9
#2 Proportion of trapped animals	19.2	2.8	3.4	3.0	24.1	25.1	48.8	20.5	9.2	30.9
#3 Proportion of night catches	50.7	64.4	55.4	91.2	58.3	85.0	68.0	82.0	75.8	77.6
#4 Proportion of day catches	49.3	35.6	44.6	8.8	41.7	15.0	32.0	18.0	24.2	22.4
#5 Mean duration of gun hunting sessions (h)	11.3	17.9	15.9	14.7	10.8	10.6	7.4	7.3	16.3	15.8
#6 Mean length of gun hunting sessions (km)	6.6	10.9	14.3	15.3	13.9	12.2	9.2	11.8	16.0	12.9
#7 Night-time activity in gun hunting (%)	39.2	44.2	44.3	69.6	45.4	53.6	46.5	56.3	49.4	55.1
**Catch composition**										
#8 Proportion of rodents	5.7	5.1	2.9	16.1	18.4	10.0	34.7	26.0	24.0	29.9
#9 Proportion of ungulates	70.0	80.4	81.4	73.4	61.4	73.0	33.9	43.8	44.6	43.2
#10 Proportion of primates	13.6	12.1	10.3	3.8	8.1	6.0	10.4	9.3	11.9	10.2
#11 Proportion of birds	1.7	0.2	0.9	0.1	3.3	0.6	2.6	2.5	2.7	2.3
#12 BlueDuiker%	27.6	46.8	34.8	54.0	38.5	55.6	60.6	50.8	48.2	49.3
#13 Mean Body Mass (kg)	12.5	9.2	16.5	9.7	10.7	11.2	6.7	6.2	6.7	6.7
**Hunter return**										
#14 Proportion of unsuccessful hunts	49	39	24	12	19	31	23	36	25	36
#15 Proportion of unsuccessful gun hunts	57	36	24	11	15	31	16	39	24	23
#16 Proportion of unsuccessful trap hunts	15	64	23	16	34	39	32	58	44	52
#17 Exploitation rate (kg.km^{-2}y^{-1})	0.19	0.11	0.12	0.16	0.26	0.32	0.22	0.19	0.19	0.21
#18 CPUE per hunting trip (kg/hunt)	8.37	13.68	23.68	21.85	16.63	21.41	13.60	8.04	10.79	11.53
#19 CPUE per hunting hour (kg/h)	1.46	1.12	2.59	1.66	2.15	1.94	2.39	1.23	1.01	1.09
#20 CPUE per hunting kilometer (kg/km)	0.44	0.64	1.31	1.33	0.99	1.09	1.65	0.61	0.46	0.66
#21 CPUE Rodents per hunting trip (kg/hunt)	0.06	0.22	0.12	1.21	0.56	0.32	0.88	0.61	1.11	0.98
#22 CPUE Rodents per hunting hour (kg/h)	0.02	0.02	0.02	0.10	0.10	0.03	0.29	0.10	0.11	0.10
#23 CPUE Rodents per hunting kilometer (kg/km)	0.00	0.02	0.01	0.08	0.03	0.02	0.08	0.06	0.06	0.08

#24 CPUE Ungulates per hunting trip (kg/hunt)	7.13	12.47	22.46	19.47	15.04	19.35	8.10	6.06	7.46	8.51
#25 CPUE Ungulates per hunting hour (kg/h)	1.12	1.01	2.43	1.46	1.90	1.70	0.87	0.78	0.65	0.78
#26 CPUE Ungulates per hunting kilometer (kg/km)	0.28	0.58	1.21	1.20	0.90	0.96	0.64	0.44	0.29	0.45
#27 CPUE Primates per hunting trip (kg)	0.54	0.85	0.73	0.38	0.58	0.81	1.39	0.54	0.86	0.98
#28 CPUE Primates per hunting hour (kg/h)	0.17	0.08	0.10	0.04	0.10	0.12	0.34	0.14	0.10	0.09
#29 CPUE Primates per hunting kilometer (kg/km)	0.13	0.04	0.07	0.03	0.04	0.07	0.27	0.05	0.07	0.05
#30 CPUE Birds per hunting trip (kg/hunt)	0.02	0.01	0.03	0.00	0.02	0.03	0.11	0.04	0.07	0.07
#31 CPUE Birds per hunting hour (kg/h)	0.00	0.00	0.00	0.00	0.00	0.00	0.03	0.01	0.01	0.01
#32 CPUE Birds per hunting kilometer (kg/km)	0.00	0.00	0.00	0.00	0.00	0.00	0.04	0.01	0.01	0.01
**Wild meat use**										
#33 Proportion of carcasses traded	49.4	61.3	47.7	89.2	51.1	54.3	83.7	84.2	91.3	87.8

Supplementary Table 4: Correlations of hunting indicators with the Bray–Curtis index. The description and assumed trend toward increasing degradation for each indicator are described (see Wildmeat database for more detailed information). Pearson correlation (*r*) with the Bray–Curtis index and associated *P* values are displayed.

Indicator	Measure	Assumptions	Indicator	*r*	*P*	$P_{adjusted}$
1) Hunting method						
Proportion of capture method	Percentage of animals shot or trapped during all hunting trips.	If the likelihood of accessing firearms for hunting remains constant, a higher incidence of trapping may be expected as the fauna becomes impoverished, as trapping allows for longer prospecting efforts. Hunters also prefer trapping smaller species because they are more challenging to shoot and provide lower biomass per cartridge. Hunters are expected to switch from gun to traps when large animals are extirpated.	#1 Proportion of shot individuals	-0.55	0.099	1
			#2 Proportion of trapped animals	0.491	0.149	1
Proportion of night and day catches	Percentage of animals shot during the night (18:26 – 05:49) or the day for strict gun hunts.	When daylight hunting, considered safer and less arduous, no longer generates a satisfactory economic return due to game depletion, hunters may resort to nocturnal hunting practices, which increasingly become the preferred practice.	#3 Proportion of night catches	0.49	0.151	1
			#4 Proportion of day catches	-0.49	0.151	1
Gun hunt characteristics	Average duration and distance and proportion night-time activity of gun hunting trips.	Increases in both time and distance travelled indicates more depleted faunas. Night-time activity is also expected to increase with game depletion as described above.	#5 Mean duration of gun hunting sessions (h)	-0.183	0.613	1
			#6 Mean length of gun hunting sessions (km)	0.301	0.399	1
			#7 Night-time activity in gun hunting (%)	0.309	0.385	1
1) Catch composition						
Proportion of hunted animal groups	Percentage of the different groups in the total number of animals hunted by all participating hunters per village.	An increase in the proportion of rodents in hunting offtake, inversely proportional to the proportion of ungulates is an indication of a more altered animal assemblage.	#8 Proportion of rodents	0.88	0.001	0.026
			#9 Proportion of ungulates	-0.886	0.001	0.020
			#10 Proportion of primates	-0.001	0.998	1
			#11 Proportion of birds	0.627	0.052	1

Percentage of blue duiker	Percentage of blue duikers against all duikers' (*Cephalophus spp*. and *Philantomba monticola*) harvests.	The contribution of the blue duiker to the total duiker harvest is expected to increase in degraded areas, as medium and large duikers are more sensitive to hunting pressure.	#12 BlueDuiker%	0.547	0.102	1
Mean Body Mass of harvest	Average body mass of an animal community, calculated as sum of total number of hunted animals per species times the body mass of each species, divided by the total number of animals hunted of all species.	A decrease in mean body mass indicates a reduction in the populations of larger (*i.e.*, less resilient) species.	#13 Mean Body Mass (kg)	-0.784	0.007	0.241
2) Hunter returns						
Unsuccessful hunting trips	Proportion of hunting trips (all types of hunts, strict gun hunts and strict trap inspection trips) that were unsuccessful.	An increase in the proportion of unsuccessful hunting trips could indicate a decline in game populations.	#14 Proportion of unsuccessful hunts	-0.122	0.738	1
			#15 Proportion of unsuccessful gun hunts	-0.325	0.36	1
			#16 Proportion of unsuccessful trap hunts	0.455	0.187	1

Species exploitation rate	Average biomass extraction per km² and per year, calculated by summing the total biomass extracted by hunters over the period monitored divided by the surface area of the hunting territory and the survey effort.	If we consider that population growth is density-dependent and follows a parabolic relationship (maximum growth at half carrying capacity), the rate of exploitation by hunters should reach its maximum in areas of intermediate degradation where the carrying capacity of game populations reaches 50%.	#17 Exploitation rate ($kg.km^{-2}y^{-1}$)	0.351	0.32	1
Catch per unit effort	Biomass of individuals harvested divided by hunting effort (*i.e.*, hunt, hours and kilometer) considering all species and species groups (rodents, ungulates, primates and birds).	A decrease in catch-per-unit-effort (CPUE) metrics indicates fewer individuals are caught in a given hunting effort, reflecting the potential reduction of game populations.	#18 CPUE per hunting trip (kg/hunt)	-0.411	0.237	1
			#19 CPUE per hunting hour (kg/h)	-0.339	0.337	1
			#20 CPUE per hunting kilometer (kg/km)	-0.135	0.71	1
			#21 CPUE Rodents per hunting trip (kg/hunt)	0.698	0.025	0.815
			#22 CPUE Rodents per hunting hour (kg/h)	0.577	0.081	1
			#23 CPUE Rodents per hunting kilometer (kg/km)	0.751	0.012	0.406
			#24 CPUE Ungulates per hunting trip (kg/hunt)	-0.531	0.115	1
			#25 CPUE Ungulates per hunting hour (kg/h)	-0.599	0.067	1
			#26 CPUE Ungulates per hunting kilometer (kg/km)	-0.428	0.217	1
			#27 CPUE Primates per hunting trip (kg)	0.445	0.198	1
			#28 CPUE Primates per hunting hour (kg/h)	0.187	0.604	1
			#29 CPUE Primates per hunting kilometer (kg/km)	0.124	0.734	1

			#30 CPUE Birds per hunting trip (kg/hunt)	0.745	0.013	0.445
			#31 CPUE Birds per hunting hour (kg/h)	0.673	0.033	1
			#32 CPUE Birds per hunting kilometer (kg/km)	0.517	0.126	1
4) Wild meat use						
Trade rate	Percentage of carcasses or pieces of carcasses sold	If hunting systems and wildmeat prices are driven by supply and demand, a decrease in the supply of game populations in hunted forests, along with a constant or even rising demand, would result in an increased sale price. Consequently, this motivates hunters to promote sales in order to generate income. The trade rate would therefore increase with the depletion of game populations.	#33 Proportion of carcasses traded	0.753	0.012	0.393

Supplementary Table 5: Potential reference assemblages for scaling up the approach across central African forests. Studies and localities where relatively intact assemblages of faunas were presumed have been recently documented.

Country	Locality	Category	Source
Cameroon	Lobeké	National Park	Line transect (Nzooh *et al*., 2016c)<br>Camera trap (Gessner *et al.*, 2013)<br>Camera trap (Poulain *et al.*, 2023)
Gabon	Ivindo	National Park	Camera-trap data (Bahaa-El-Din *et al*., 2016)<br>Line transect (Maisels *et al*., 2010)<br>Other inventory reviewed (Van Vliet *et al*., 2007)
	Cora Wood	Forestry Concession	Camera-trap data (Bahaa-El-Din *et al*., 2016)
	Precious Wood	Forestry Concession	Camera-trap data (Bahaa-El-Din *et al*., 2016)
	Lopé	National Park	Camera-trap data (Bahaa-El-Din *et al*., 2016)
	Moukalaba-Doudou	National Park	Camera-trap data (Bahaa-El-Din *et al*., 2016)
Central African Republic	Dzanga–Sangha	National Park/Special Reserve	Line-transect (Princée, 2013)
	SINFOCAM	Forestry Concession	Camera-trap data (Beudels-Jamar *et al*., 2016)
Republic of Congo	Nouabalé-Ndoki	National Park	Camera-trap data (Gessner *et al*., 2014)<br>Camera-trap data (Breuer *et al.*, 2021)<br>Line-transect (Stokes *et al*., 2010; Poulsen *et al*., 2011; Maisels *et al*., 2012)
	Odzala-Kokoua	National Park	Camera-trap data (Henschel *et al*., 2014)<br>Line transect (Maisels *et al.*, 2013)
Democratic Republic of Congo	Salonga	National Park	Camera-trap data (Bessone *et al*., 2020)
